# Identification of prolactin receptor variants with diverse effects on receptor signalling

**DOI:** 10.1530/JME-22-0164

**Published:** 2023-01-25

**Authors:** Caroline M Gorvin, Paul J Newey, Rajesh V Thakker

**Affiliations:** 1 Academic Endocrine Unit, Oxford Centre for Diabetes, Endocrinology and Metabolism, Radcliffe Department of Medicine, University of Oxford, Oxford, UK; 2 Oxford NIHR Biomedical Research Centre, University of Oxford, Churchill Hospital, Oxford, UK; 3 Institute of Metabolism and Systems Research (IMSR) & Centre for Endocrinology, Diabetes and Metabolism (CEDAM), Birmingham Health Partners, University of Birmingham, Birmingham, UK; 4 Centre of Membrane Proteins and Receptors (COMPARE), University of Birmingham, Birmingham, UK; 5 Division of Molecular & Clinical Medicine (MCM), University of Dundee, Jacqui Wood Cancer Centre, Dundee, UK

**Keywords:** hyperprolactinaemia, prolactinoma, proliferation, JAK-STAT5

## Abstract

The prolactin receptor (PRLR) signals predominantly through the JAK2-STAT5 pathway regulating multiple physiological functions relating to fertility, lactation, and metabolism. However, the molecular pathology and role of PRLR mutations and signalling are incompletely defined, with progress hampered by a lack of reported disease-associated PRLR variants. To date, two common germline *PRLR* variants are reported to demonstrate constitutive activity, with one, Ile146Leu, overrepresented in benign breast disease, while a rare activating variant, Asn492Ile, is reported to be associated with an increased incidence of prolactinoma. In contrast, an inactivating germline heterozygous *PRLR* variant (His188Arg) was reported in a kindred with hyperprolactinaemia, while an inactivating compound heterozygous *PRLR* variant (Pro269Leu/Arg171Stop) was identified in an individual with hyperprolactinaemia and agalactia. We hypothesised that additional rare germline *PRLR* variants, identified from large-scale sequencing projects (ExAC and GnomAD), may be associated with altered *in vitro* PRLR signalling activity. We therefore evaluated >300 previously uncharacterised non-synonymous, germline *PRLR* variants and selected 10 variants for *in vitro* analysis based on protein prediction algorithms, proximity to known functional domains and structural modelling. Five variants, including extracellular and intracellular domain variants, were associated with altered responses when compared to the wild-type receptor. These altered responses included loss- and gain-of-function activities related to STAT5 signalling, Akt and FOXO1 activity, as well as cell viability and apoptosis. These studies provide further insight into PRLR structure–function and indicate that rare germline *PRLR* variants may have diverse modulating effects on PRLR signalling, although the pathophysiologic relevance of such alterations remains to be defined.

## Introduction

The prolactin receptor (PRLR), and its ligand, the hormone prolactin (PRL) are reported to have diverse roles that include induction and maintenance of lactation in the peripartum and postpartum periods (Ben-Jonathan *et al.* 2008), parental behaviour, immune function, reproduction and metabolic functions such as pregnancy-related increases in β-cell mass, and regulation of lipid content and body temperature (Freemark *et al.* 2002, Schuff *et al.* 2002, Viengchareun *et al.* 2004, Viengchareun *et al.* 2008, Huang *et al.* 2009, Banerjee *et al.* 2016, Smiley *et al.* 2022). PRL binds to the PRLR, a class I cytokine receptor, which is functionally active as a homodimer (Gadd & Clevenger 2006, Qazi *et al.* 2006, Brooks *et al.* 2014). Each mature PRLR monomer has a multi-domain structure consisting of a highly conserved ligand-binding extracellular domain (ECD, 1–210), a transmembrane α-helix (TM, residues 211–234), and an intracellular domain (ICD, residues 235–598). Structural analysis of the ECD has revealed two subdomains, designated D1 and D2, which are important in ligand binding and subsequent receptor activation (Svensson *et al.* 2008, Broutin *et al.* 2010, van Agthoven *et al.* 2010, Rao & Brooks 2011, Brooks 2012), and the WSxWS motif, which acts as a molecular switch during activation (Broutin *et al.* 2010, van Agthoven *et al.* 2010, Dagil *et al.* 2012).

The ICD interacts with the Janus kinase-2 (JAK2) protein that associates with a highly conserved structural motif named Box 1 (residues 243–251) and potentially, with a second motif, Box 2 (residues 287–296) within the ICD (Lebrun *et al.* 1995). Moreover, recent structural studies have revealed that both PRLR and the related growth hormone receptor (GHR) harbour conserved regions that interact with lipids, referred to as lipid-interacting domains or LIDs within the inner plasma membrane leaflet thereby allowing a greater surface area and potentially, simultaneous interaction with multiple signalling kinases (Haxholm *et al.* 2015, Bugge *et al.* 2016). Hormone binding to the ECD activates conformational changes within the TMD and ICD, allowing separation of the ICDs and initiating phosphorylation cascades downstream of JAK2 (Brown *et al.* 2005, Brooks *et al.* 2014, Haxholm *et al.* 2015, Bugge *et al.* 2016). JAK2 activates complex signalling pathways, predominantly via interaction with the signal activator of transcription 5 (STAT5) pathway (Brooks 2012), and also by the phosphatidylinositol 3-kinase (PI3K)/Akt and mitogen-activated protein kinase pathways (Fresno Vara *et al.* 2000, Amaral *et al.* 2004, Brooks 2012). These signal pathways lead to transcription of target genes that regulate proliferation, differentiation, and cell survival (Fresno Vara *et al.* 2000, Amaral *et al.* 2004, Brooks 2012). Despite these findings, the function of individual residues in receptor activation and signal transduction is poorly understood.

A number of human studies have highlighted residues that are important for receptor function and subsequent signal transduction. The PRLR variants Ile76Val and Ile146Leu were reported to be gain-of-function variants with constitutive activity that occur in 15% of women with breast fibroadenomas (Bogorad *et al.* 2008, Courtillot *et al.* 2010). However, more recent studies did not detect such correlations (Glasow *et al.* 2001, Vaclavicek *et al.* 2006, Lee *et al.* 2007, Nyante *et al.* 2011) or marked changes in signalling activity (Bernard *et al.* 2016, Gorvin *et al.* 2018b), although the Ile146 residue has been shown to be important for receptor folding and stability (Dagil *et al.* 2012, Zhang *et al.* 2015). A loss-of-function pathogenic germline *PRLR* variant (His188Arg), which affected a highly conserved His188 residue within the D2 domain that is important for hormone binding, was described in a family with hyperprolactinaemia (Kulkarni *et al.* 2010, Newey *et al.* 2013). Subsequently, an individual with hyperprolactinaemia and agalactia was reported with germline compound heterozygous nonsense (Arg171Stop) and missense (Pro269Leu) PRLR variants (Kobayashi *et al.* 2018), and recently, a germline Asn492Ile PRLR variant that increases receptor activity via the PI3K-Akt pathway was reported to be associated with a higher incidence of prolactinoma (Gorvin *et al.* 2018b).

Both the Ile146Leu and Asn492Ile variants are present in recently described population databases such as the Exome Aggregation Consortium (ExAC) and the Genome Aggregation Database (GnomAD) (Karczewski *et al.* 2020), and we hypothesised that a further examination of these population-based databases could yield important structural and functional insights for individual PRLR residues and provide information on activation of specific signalling pathways. Indeed, similar studies have previously identified residues within the adaptor protein-2 sigma subunit that are important for calcium homeostasis (Gorvin *et al.* 2018a) and residues in α-N-acetylglucosaminidase that contribute to the rare lysosomal storage disease Sanfilippo type-B (Clark *et al.* 2018). We therefore examined the ExAC/GnomAD datasets with the aim of identifying missense coding variants in the PRLR, which could be characterised by their functional consequences.

## Materials and methods

### Protein sequence alignment and three-dimensional modelling of PRLR structure

The population frequencies of germline non-synonymous *PRLR* single nucleotide variants (SNVs) were evaluated using ExAC and Genome Aggregation Databases (both datasets (ExAC and GnomAD v.2.1) now reported at GnomAD (https://gnomad.broadinstitute.org/) (Karczewski *et al.* 2020)). SIFT, MutationTaster, Polyphen-2, and REVEL were used to predict the effect of amino acid substitutions (Kumar *et al.* 2009, Adzhubei *et al.* 2010, Schwarz *et al.* 2014, Ioannidis *et al.* 2016). Amino acid conservation was examined in PRLR mammalian orthologs using ClustalW2 (Larkin *et al.* 2007). The crystal structure of the two chains of the rat PRLR extracellular domain in complex with PRL (Protein Data Bank (PDB) accession code 3NPZ) and the NMR structure of the human PRLR ECD D2 domain (PDB:2LFG) (van Agthoven *et al.* 2010, Dagil *et al.* 2012) were used to predict the effect of ECD variants on PRLR structure. The NMR structure of the single-pass transmembrane domain of PRLR (PDB:2N7I) (Bugge *et al.* 2016) was used to predict the structural effect of TMD variants. Figures were prepared using the PyMOL Molecular Graphics System (Schrödinger, New York, NY, USA).

### Cell culture and transfection

PRLR variants were introduced into the wild-type (WT) pd*EYFP-PRLR* construct by site-directed mutagenesis using the Quikchange Lightning Kit (Agilent Technologies) and gene-specific primers (Sigma) and were confirmed as previously described (Newey *et al.* 2013). Expression constructs were transiently transfected into HEK293 cells and were maintained in DMEM-Glutamax media (Gibco) with 10% fetal bovine serum (Gibco) at 37ºC, 5% CO_2_, using Lipofectamine 2000 (LifeTechnologies), as described (Newey *et al.* 2013), and functional studies were performed using poly-l-lysine-treated plates. Western blot analysis was used to assess the expression of transfected PRLR and endogenous α-tubulin as a loading control, using anti-PRLR (1:1000, Santa Cruz Biotechnology) and anti-α-tubulin (1:1000, Abcam) antibodies, as described (Newey *et al.* 2013).

### Confocal microscopy

Confocal imaging was performed as previously described (Gorvin *et al.* 2018b). Cells were plated in six-well plates containing poly-l-lysine-treated coverslips and cultured at 37°C. Cells were transiently transfected with 1000 ng of either WT or variant PRLR expression constructs. After 24 h, cells were fixed in 4% paraformaldehyde/PBS (Sigma-Aldrich), permeabilised in 1% Triton-X100/PBS (Thermo Scientific), and immunostained with primary anti-PRLR (1:200, Santa Cruz Biotechnology) and secondary antibody Alexa Fluor-488 (1:1000, Molecular Probes). Cells were mounted in Prolong Gold Antifade reagent (Invitrogen). Images were captured using a Zeiss LSM780 confocal microscope with a Plan-Apochromat x63/1.2/water DIC objective. An argon laser (488 nm) was used to excite Alexa Fluor-488.

### AlphaScreen SureFire assays

AlphaScreen assays were performed as previously described (Newey *et al.* 2013, Gorvin *et al.* 2018b). Cells were transiently transfected in 48-well plates with 200 ng of either WT or variant PRLR vectors. After 30 h, cells were incubated in serum-free media for 12 h prior to treatment with human recombinant PRL (PromoCell, Heidelberg, Germany) for 20 min at concentrations ranging from 0 to 1000 ng/mL. Cells were lysed in Surefire lysis buffer, and AlphaScreen Surefire pSTAT5 or pAkt assays (PerkinElmer) were performed according to manufacturer’s instructions (Binder *et al.* 2008). The fluorescence signal was measured using a PHERAstar *FS* microplate reader (BMG Labtech, Aylesbury, UK). A minimum of four independently transfected replicates were used for each construct within each experiment, and each experiment was performed on four to five separate occasions with different cell passages. Data were plotted as fold-change responses relative to the response at 0 ng/mL in cells expressing the WT PRLR expression construct. Statistical analyses were performed using two-way ANOVA with Dunnett’s or Tukey’s multiple-comparison tests for pSTAT5 studies and by one-way ANOVA with Sidak’s multiple-comparison tests for pAkt studies

### Luciferase reporter assays

The Forkhead box O1 (*FOXO1*) promoter region was PCR amplified from human genomic DNA using previously described primers (Essaghir *et al.* 2009) and cloned into the pGL4.10 vector (Promega). The sequence of the insert was confirmed by Sanger DNA sequencing (Source Bioscience, Nottingham, UK). The pGL4.10 vector containing the cytokine-inducible SH2-containing protein (*CISH*) reporter has been described previously (Newey *et al.* 2013). HEK293 cells were transiently co-transfected in 24-well plates with 100 ng of pGL4.10-CISH reporter gene construct, 10 ng of PRL (renilla) control vector, and 100 ng of WT or variant PRLR vectors. Following transfection, cells were incubated in serum-free media overnight. Cells were then treated with 0–500 ng/mL PRL for 24 h in serum-free media. Cells were lysed and assayed for luciferase activity using a Turner Biosystems (Promega) or Centro LB960 (Berthold Technologies, Harpenden, Hertfordshire, UK) luminometer and the Dual-Luciferase Reporter assay system (Promega). The firefly luciferase activity was adjusted for Renilla luciferase activity (Firefly/Renilla ratio) and ratios were expressed as a fold-change relative to cells treated with 0 ng/mL of PRL within each group. A minimum of four independently transfected replicates were performed in each experiment, and each experiment was performed on four to seven separate occasions with different cell passages. Statistical analysis was performed by two-way ANOVA with Sidak’s or Dunnett’s multiple-comparisons test for *CISH* and by Kruskal–Wallis with Dunn’s test or one-way ANOVA with Dunnett’s test for *FOXO1*.

### Cell viability assay

Cells were plated in 96-well plates and transfected with 50 ng of WT or variant PRLR per well. Following 24 h, cells were treated with 200 ng/mL of PRL and cell viability was assessed 96 h later using the CellTiter Blue kit (Promega) (Gorvin *et al.* 2018b). The cell count for day 1 (i.e. time 0 before PRL was added) was set as 1 and each cell count was expressed relative to this original cell count. Plates were read on a PHERAstar *FS* microplate reader (BMG Labtech). A minimum of four independently transfected replicates were performed in each experiment, and each experiment was performed on four separate occasions with different cell passages. Statistical analysis was performed by one-way ANOVA with Dunnett’s test or Kruskal–Wallis with Dunn’s test.

### Apoptosis assay

Cells were plated in 96-well plates and transfected with 50 ng of WT or variant PRLR vectors per well. Following 24 h, cells were treated with 0 ng/mL or 200 ng/mL of PRL and apoptosis was assessed at 0 and 96 h post-PRL treatment using the Caspase-Glo-3/7 kit (Promega) (Gorvin *et al.* 2018b). Plates were read on a Centro LB960 luminometer. A minimum of four independently transfected replicates were performed in each experiment, and each experiment was performed on four to six separate occasions with different cell passages. Statistical analysis was performed by one-way ANOVA with Dunnett’s multiple comparisons test.

### Statistics

The number of experimental replicates denoted by *n* is indicated in each figure legend. Data were plotted and statistical analyses were performed in Graphpad Prism 7. Normality tests (Shapiro–Wilk or D’Agostino–Pearson) were performed on all datasets to determine whether parametric or non-parametric tests were appropriate. A *P*-value of < 0.05 was considered statistically significant. A minimum of four independently transfected replicates was performed in all cell-based assays, and each experiment was performed on separate occasions with different passages of cells. Specific details of each test are outlined in the figure legends and within the relevant methods section.

## Results

### Identification of non-synonymous PRLR variants and their predicted effects on protein function

An analysis of the ExAC (v1.0) and GnomAD (v2.1.1) databases was performed to identify non-synonymous, missense *PRLR* variants in the full-length, membrane-expressed (i.e. excluding the 24 amino acid signal peptide) protein. These analyses revealed 310 non-synonymous missense *PRLR* variants comprising 85 ECD variants, 10 TMD variants, and 215 ICD variants. The distribution of variants between the ECD, TMD, and ICD was 27.4, 3.2, and 69.4%, respectively. This was significantly different from that expected based on the size of each region (*P* < 0.05, χ^2^-test), with fewer variants observed in the ECD than expected (37.7%), indicating this region may be less tolerant to variation.

The predicted deleteriousness/pathogenicity of each PRLR variant was determined by assessing their population frequency; their effect on protein function using four online prediction software packages (SIFT, Polyphen-2, MutationTaster, and REVEL (Kumar *et al.* 2009, Adzhubei *et al.* 2013, Schwarz *et al.* 2014, Ioannidis *et al.* 2016)) and the evolutionary conservation of each residue in mammalian species. Following exclusion of variants that have previously been functionally expressed (e.g. Gly57Ser, Ile76Val, Ile146Leu, Gly376Gln, Asn492Ile, and Glu554Gln (Bogorad *et al.* 2008, Newey *et al.* 2013, Bernard *et al.* 2016, Gorvin *et al.* 2018b)), examination of the minor allele frequencies (MAF) of all the remaining variants revealed them to be rare (defined as a MAF of <1% (Agarwala *et al.* 2013)). Variants that were predicted benign or tolerated by all the prediction programs, and those that affected residues that were conserved in fewer than two mammalian orthologues, were excluded from further analyses. Thus, 42 ECD, 5 TMD, and 93 ICD variants (Table 1) were predicted to be potentially deleterious/pathogenic by these criteria, indicating that they may have functional consequences on PRLR signalling.

### Structural characterisation of the ECD PRLR variants

**Figure 1:**
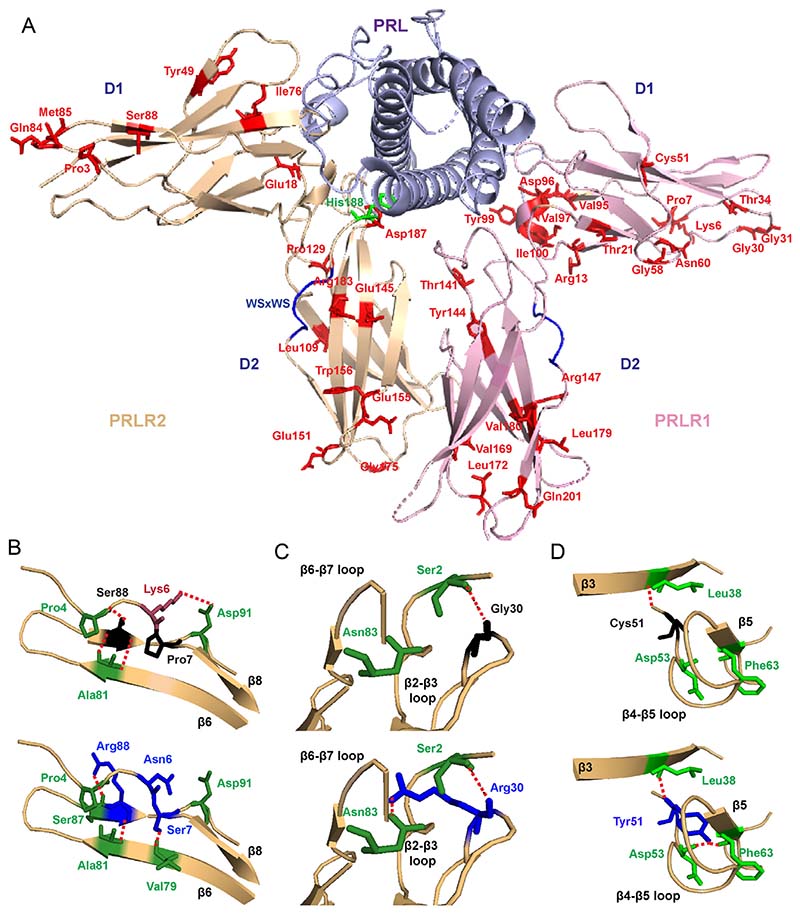
Location of the PRLR ECD variants and structural characterisation of rare variants located in D1. (A) Crystal structure showing the two monomers (PRLR1 in pink and PRLR2 in light brown) of the rat PRLR extracellular domain (ECD) in complex with the PRL hormone (purple) (PDB 3NPZ (van Agthoven *et al.* 2010)). Each PRLR ECD monomer is comprised of two subdomains, designated D1 (residues 1–101) and D2 (residues 109–210), which are important in ligand binding and subsequent receptor activation (Svensson *et al.* 2008, Broutin *et al.* 2010, van Agthoven *et al.* 2010, Rao and Brooks 2011, Brooks 2012). Side chains of the potential deleterious variant residues from ExAC and GnomAD are shown in red. The His188 residue that is mutated in hyperprolactinaemia is shown in green. Residue 49 is labelled as Tyr in the rat structure, and in humans, this is His49. Residue 169 is labelled as Val in the rat structure, and in human, this is Ile169. Rare variants are shown in only one monomer. (B) Lys6, Pro7, and Ser88 are located in close proximity within the β6-β7-β8 region (Top). The variant Asn6 loses contact with Asp91 on the β8 strand, Ser7 gains contact with Val79 on the β6 strand, and Arg88 loses contact with Pro4 and gains a new contact with the adjacent Ser87 (all blue, bottom). (C) The Gly30 residue, located in the β2-β3 loop, forms a polar contact with the Ser2 residue (Top). When mutated to Arg30 (blue, bottom), the longer side chain is able to retain this contact and form a new hydrogen bond with Asn83 in the β6-β7 loop. (D) The Cys51 residue is located in the β4-β5 loop. Mutation in Tyr51 (blue, bottom) adds a more bulky residue within this tightly packed region, which is predicted to result in gain of two contacts with Asp53 and Phe63 of the β4-β5 loop.

**Figure 2:**
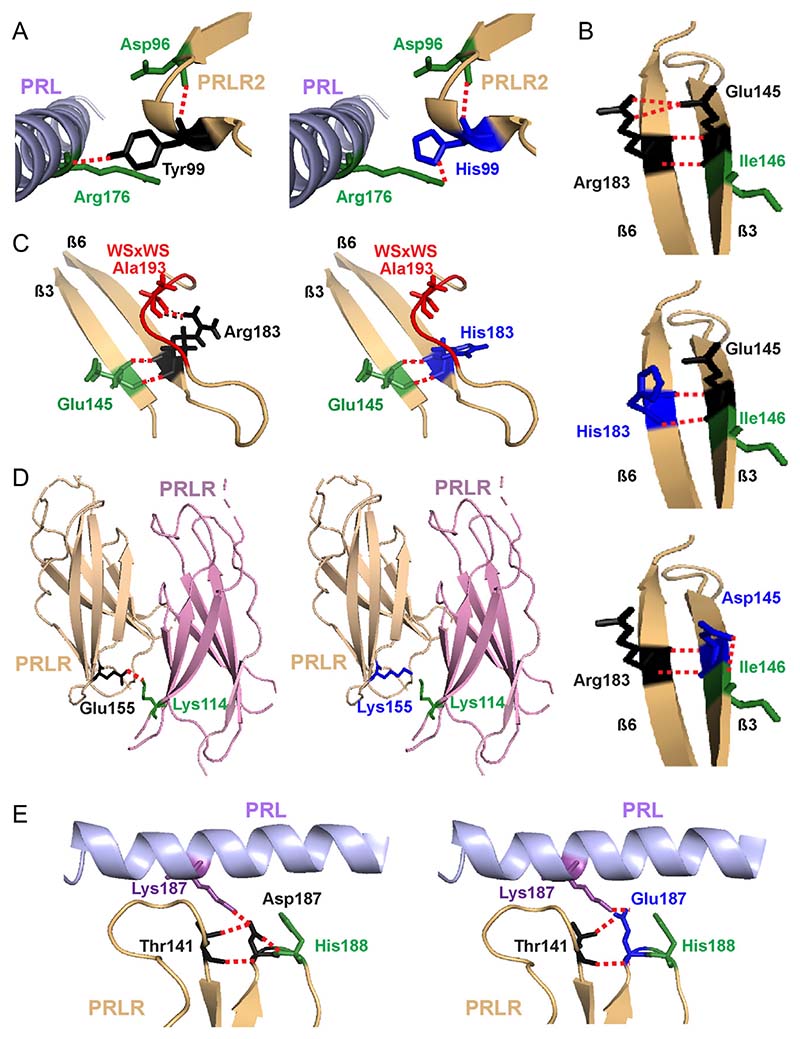
Structural characterisation of rare variants located in the C-terminal D1 and D2 of PRLR ECD. (A) The Tyr99 residue is located close to the PRL-binding site with the PRLR. The hydroxyl group of the wild-type Tyr99 contacts Arg176 on the PRL protein (left). Mutation to His99 (blue, right) predicts retention of the contact, but at a more distal site, further away from the PRL α-helix and therefore is likely to increase the distance between the PRL and PRLR molecules which may affect binding and activation. (B) The Glu145 and Arg183 PRLR residues are located in adjacent β-strands and form polar contacts with each other. Mutation to His183 (middle) and Asp145 (bottom) is predicted to disrupt two of these contacts. Additionally, Asp145 is predicted to lose contact with the neighbouring Ile146, a residue previously demonstrated to be important for PRLR folding and stability (Dagil *et al.* 2012, Zhang *et al.* 2015). (C) The His183 also loses contact with Ala193, which forms part of the highly conserved WSxWS motif (red). (D) The wild-type Glu155 forms a contact with Lys114 on the opposite PRLR protomer. The Lys155 variant loses this contact and may disrupt homodimeric structural stability. (E) The wild-type Asp187 residue lies close to the PRL binding site and forms a contact with the His188 residue of PRLR, which has a critical role in ligand binding (Kulkarni *et al.* 2010) (Left). Mutation to Glu187 (blue, right) leads to loss of this contact, which may affect activation of the PRLR protein.

Three-dimensional modelling using the crystal structure of the PRL-bound homodimeric ECD and the NMR structure of the ligand-free D2 domain (van Agthoven *et al.* 2010, Dagil *et al.* 2012) was performed to determine the locations of the ECD PRLR variants and predict their effects on structural integrity. This revealed that 23 of the total 42 variants were within the D1 lobe and the remaining 19 variants were within the D2 lobe, of which 11 variants (6 in D1 and 5 in D2) were predicted to have structural effects on the PRLR protein (Table 1, Fig. 1A). Three variants (Pro7Ser, Gly30Arg, Cys51Tyr) are predicted to gain contacts with adjacent residues, six variants (Lys6Asn, Tyr99His, Thr141Met, Glu155Lys, Arg183His, Asp187Glu) are predicted to lose contacts with adjacent residues, and two variants (Ser88Arg, Glu145Asp) are predicted to both lose and gain contacts with adjacent PRLR residues (Fig. 1B, C, D and 2, Table 1). These predicted changes may affect ECD flexibility and disrupt PRLR activation.

Several variants were predicted to affect important structural components in the C-terminal region of D1 and in D2 of the PRLR ECD (Fig. 2). The Tyr99His variant, in D1, is predicted to retain contact with the PRL ligand but at a more distal site (i.e further away from the PRL α-helix) and therefore is likely to increase the distance between the PRL and PRLR molecules, which may affect agonist binding or PRLR activation (Fig. 2A, Table 1). Two D2 variant residues, His183 and Asp145, are predicted to disrupt contacts between the wild-type Arg183 and Glu145 residues. The Glu145 and Arg183 PRLR residues are located in adjacent β-strands and form four direct contacts (Fig. 2B). The variants Asp145 and His183 are predicted to disrupt two of these contacts and may increase the flexibility of the D2 lobe (Fig. 2B). Additionally, the Asp145 variant is predicted to lose contact with the neighbouring Ile146, a residue previously demonstrated to be important for PRLR folding and stability (Dagil *et al.* 2012, Zhang *et al.* 2015) (Fig. 2B); while His183 loses contact with Ala193, a residue that forms part of the highly-conserved WSxWS motif (Fig. 2C). Arg183 is one of five highly conserved residues (Ile146, Glu151, Glu155, Tyr178, Arg183) that together with residues of the Trp-Arg ladder undergo conformational changes to switch PRLR to an active state (Dagil *et al.* 2012). Rare variants were identified to affect two more of these five residues (Glu151Lys, Glu155Lys) (Table 1). Moreover, the variant Lys155 loses contact with Lys114 on the adjacent PRLR protomer, which may affect receptor dimerisation and protein stability (Fig. 2D, Table 1). The Asp187Glu variant lies close to the PRL binding site and is predicted to lose contact with the adjacent His188 residue, which has a critical role in ligand binding (Kulkarni *et al.* 2010) and is mutated in some individuals with hyperprolactinaemia (Newey *et al.* 2013) (Fig. 2E, Table 1). Thus, the PRLR D2 domain mutations are predicted to affect PRL binding, flexibility of the PRLR structure, and PRLR activation.

### Structural characterisation of the TMD and ICD PRLR variants

**Figure 3:**
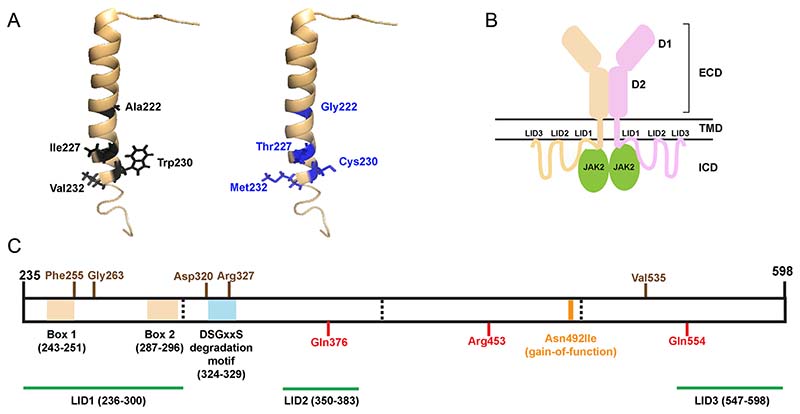
Structural characterisation of the PRLR variants located in the TMD and ICD. (A) A single α-helix forms the transmembrane domain (TMD) of the PRLR. Five rare PRLR variants (Ala222Gly, Ile227Thr, Trp230Cys, Val232Ala, Val232Met) are located within the TMD, and four of these are present within the published NMR structure of the PRLR TMD (PDB:2N7I (Bugge *et al.* 2016)). The four WT residues (black) are located at the cytoplasmic end of the TMD and each forms backbone contacts with adjacent residues within the α-helix. The mutant residues (blue) are not predicted to affect these backbone contacts. (B) Cartoon depicting the PRLR structure with the two monomers shown in brown and pink. The extracellular domain (ECD) contains two domains (D1 and D2) and is connected to the intracellular domain (ICD) via the TMD. The ICD is predicted to interact with the plasma membrane in at least three regions known as lipid-interacting domains (LID1-3). The ICD also interacts with the JAK2 proteins that activate signalling downstream of the PRLR. (C) Cartoon showing the known functional domains of the PRLR ICD, with the amino acid residues (236–300, 243–251, 324–329, 350–383, and 547–598) involved shown in parentheses. The LIDs are shown in green. Two regions, Box 1 and Box 2 (brown-shaded regions), are binding sites for JAK2 (Lebrun *et al.* 1995), and the DSGxxS region (blue-shaded region) acts as a degradation motif (Plotnikov *et al.* 2009). The PRLR residues investigated in this study are shown in brown above the cartoon and residues investigated in previous studies are shown in red below the cartoon (Bernard *et al.* 2016, Gorvin *et al.* 2018a). The location of the Asn492Ile gain-of-function PRLR variant that is associated with prolactinoma is indicated in orange.

The NMR structure of the single-pass transmembrane domain of the PRLR (PDB:2N7I (Bugge *et al.* 2016)) was used to study the structural effects of the five highly conserved TMD PRLR variants (Ala222Gly, Ile227Thr, Trp230Cys,Val232Ala, Val232Met) that are predicted to be potentially deleterious/pathogenic (Fig. 3A). Three-dimensional modelling did not predict any changes in interactions between the TMD located PRLR variants. However, the side chains of each amino acid project into the plasma membrane bilayer, and therefore, the variant residues may affect interactions with lipids, which cannot be predicted using the current structural models. In addition to the TMD, plasma membrane interactions also occur with three regions of the ICD (LID1–3) (Bugge *et al.* 2016) (Fig. 3B). About 45 PRLR rare variants affect residues within these LID regions, 31 in LID1, 3 in LID2, and 11 in LID3 (Table 1). The ICD contains two other structural features, Box 1 and 2, the binding sites for JAK2 (Lebrun *et al.* 1995) and a conserved degradation motif, DSGxxS (located at residues 324–329) (Plotnikov *et al.* 2009) (Fig. 3C). Seven rare variants were identified in residues within Box 1, and two variants within Box 2, while three variants within the same residue were identified in the degradation motif.

### Functional analysis of the PRLR rare variants

Based on their predicted effects on pathogenicity, evolutionary conservation, and structure, we chose to assess ten PRLR rare variants. Five ECD variants (Tyr99His, Glu145Asp, Glu155Lys, Arg183His, Asp187Gly) (Fig. 2A, B, C, D and E) were selected including ones within the PRL-binding region, in the homodimeric interface and close to the WSxWS motif and five ICD variants including, two residues within LID1 located between Box 1 and 2 (Phe255Ser, Gly263Asp); two residues close to or within the degradation motif (Asp320Tyr, Arg327Gln); and one distal rare variant close to LID3 (Val535Met) (Fig. 3C).

### Effect of rare variants on PRLR expression

**Figure 4:**
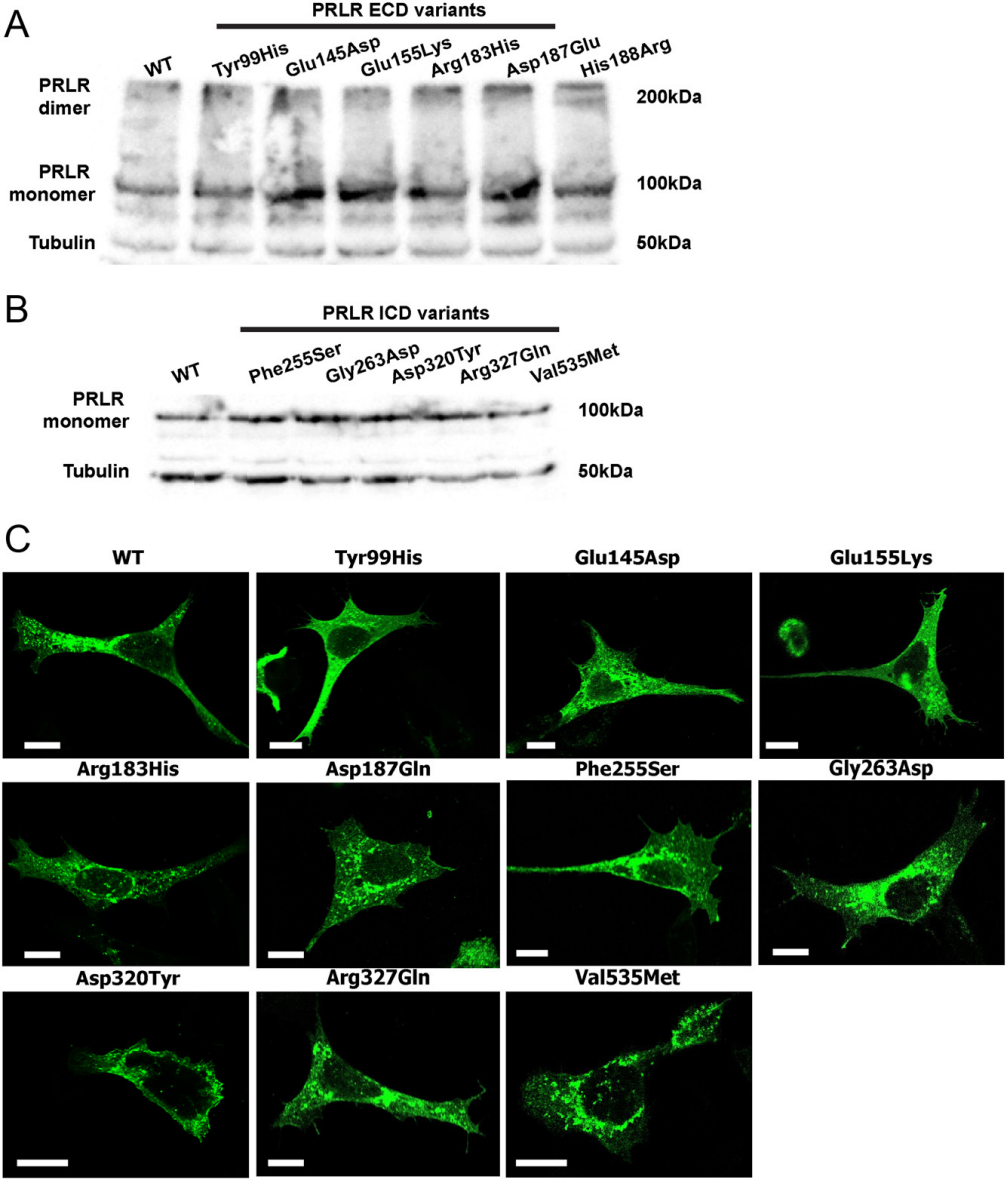
Expression of the PRLR ECD and ICD variants. Western blot analyses of HEK293 cells expressing (A) PRLR ECD rare variants and (B) PRLR ICD rare variants. Lysates show approximately equal expression levels of PRLR in cells transfected with each rare variant and the WT PRLR. Tubulin was used as a loading control. (C) Confocal microscopy images of the PRLR WT and variant proteins in transfected HEK293 cells. Bar indicates 10 µm.

Initially, PRLR protein expression was assessed by Western blot analyses in HEK293 cells transiently transfected with WT or variant PRLR (Fig. 4A and B). The PRLR protein was expressed at equivalent levels in cells transfected with the five ECD and five ICD rare variants and WT PRLR constructs (Fig. 4A and B), indicating it is unlikely that any of the variants affect protein folding. Although structural analysis had predicted that the Glu155Lys variant may affect contacts between the two PRLR protomers, concentrations of PRLR dimers were similar in cells transfected with WT or the ECD variants (Fig. 4A). Therefore, the Glu155Lys variant may not have a major impact on homodimer stability.

The cellular expression of each of the PRLR variants was then assessed using confocal microscopy. As previously observed, the WT PRLR protein is located within the cytoplasm and at the plasma membrane (Gorvin *et al.* 2018b). A similar expression pattern was observed for the ten PRLR rare variants (Fig. 4C). Therefore, it is unlikely that the variant residues affect protein expression or trafficking of the PRLR to the cell surface.

### Effect of the PRLR rare variants on STAT5 signalling

**Figure 5:**
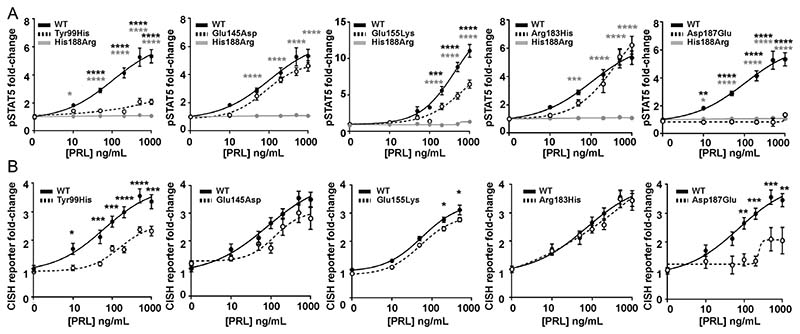
Functional characterisation of the JAK-STAT signalling pathway by PRLR ECD variants. (A) pSTAT5 responses following prolactin (PRL) treatment in cells expressing wild-type (WT), mutant His188Arg, or ECD variants Tyr99His, Glu145Asp, Glu155Lys, Arg183His, Asp187Glu. PRL-induced pSTAT5 production was abolished in His188Arg- and Asp187Glu-expressing cells and significantly reduced in Tyr99His- and Glu155Lys-expressing cells compared to cells expressing WT. (B) CISH luciferase reporter activity in cells transfected with WT or the five ECD variant PRLRs. CISH reporter activity was significantly reduced in Tyr99His-, Glu155Lys-, and Asp187Glu-expressing cells compared to WT cells. Mean from four to five independent assays for all panels. Statistical analyses show comparisons between WT and the five ECD PRLR variants (black) and WT and the His188Arg mutant (grey) by two-way ANOVA with Dunnett’s or Sidak’s multiple comparisons tests. ****P < 0.0001, ***P < 0.001, **P < 0.01, *P < 0.05.

Previous studies have demonstrated PRLR to predominantly signal via the STAT5 pathway (Brooks 2012) and we therefore assessed this pathway by measuring immediate signalling by phospho-STAT5 (pSTAT5) and later downstream effects on transcription by measuring the STAT5 target gene *CISH*. The effects on PRLR signalling were assessed together with that of the His188Arg mutant PRLR that has been reported to result in a loss of function in association with familial hyperprolactinaemia (Newey *et al.* 2013, Bernard *et al.* 2016). We first assessed the ECD variants and demonstrated that increasing concentrations of PRL led to an increase in pSTAT5 and CISH luciferase reporter in a similar dose-dependent manner in cells expressing the PRLR variants Glu145Asp and Arg183His and cells expressing WT PRLR (Fig. 5A and B). However, responses were significantly reduced in cells expressing the PRLR ECD variants Tyr99His, Glu155Lys, and Asp187Glu, when compared to those expressing WT PRLR (Fig. 5A and B, Table 2).

**Figure 6:**
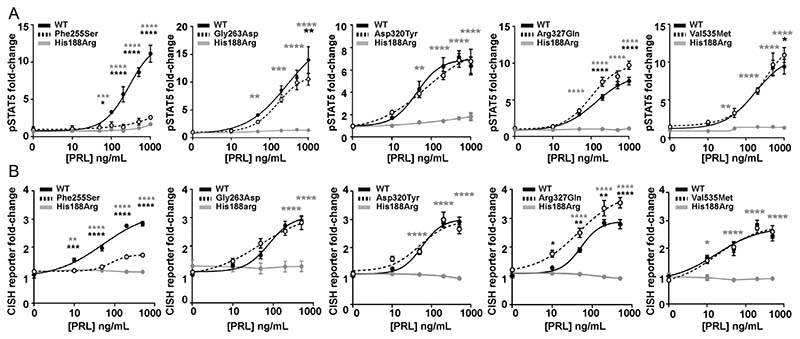
Functional characterisation of the JAK2-STAT5 signalling pathway by PRLR ICD variants. (A) pSTAT5 responses following prolactin (PRL) treatment in cells expressing wild-type (WT), mutant His188Arg, or ICD variants Phe255Ser, Gly263Asp, Asp320Tyr, Arg327Gln, Val535Met. PRL-induced pSTAT5 production was abolished in His188Arg expressing cells, significantly reduced in Phe255Ser and significantly increased in Arg327Gln expressing cells when compared to WT cells. Additionally, pSTAT5 was reduced in Gly263Asp- and Val535Met-expressing cells when treated with high (1000 ng/mL) PRL. (B) CISH luciferase reporter activity in cells transfected with WT, mutant His188Arg, or the five ICD variant PRLRs. CISH reporter activity was abolished in His188Arg-expressing cells, significantly reduced in Phe255Ser and significantly increased in Arg327Gln-expressing cells when compared to WT cells. Mean from four to five independent assays for all panels. Statistical analyses show comparisons between WT and the five ICD PRLR variants (black) and WT and the His188Arg mutant (grey) by two-way ANOVA with Dunnett’s or Tukey’s multiple comparisons tests. **** *P* < 0.0001, *** *P* < 0.001, ** *P* < 0.01, * *P* < 0.05.

The effects of the five PRLR ICD rare variants were next assessed on STAT5 signalling. Increasing concentrations of PRL led to an increase in pSTAT5 and CISH luciferase reporter in a similar dose-dependent manner in cells expressing the PRLR rare variants Gly263Asp, Asp320Tyr, and Val535Met and cells expressing WT PRLR (Fig. 6A and B). In contrast, the Phe255Ser rare ICD PRLR variant significantly reduced pSTAT5 and CISH reporter responses, while the Arg327Gln variant had significantly elevated pSTAT5 and CISH reporter responses, when compared to WT-expressing cells (Fig. 6A and B, Table 2). There was consistently no response in cells expressing the His188Arg-mutant protein to increasing concentrations of PRL (Fig. 6, Table 2).

### Effect of the PRLR rare variants on Akt signalling

**Figure 7:**
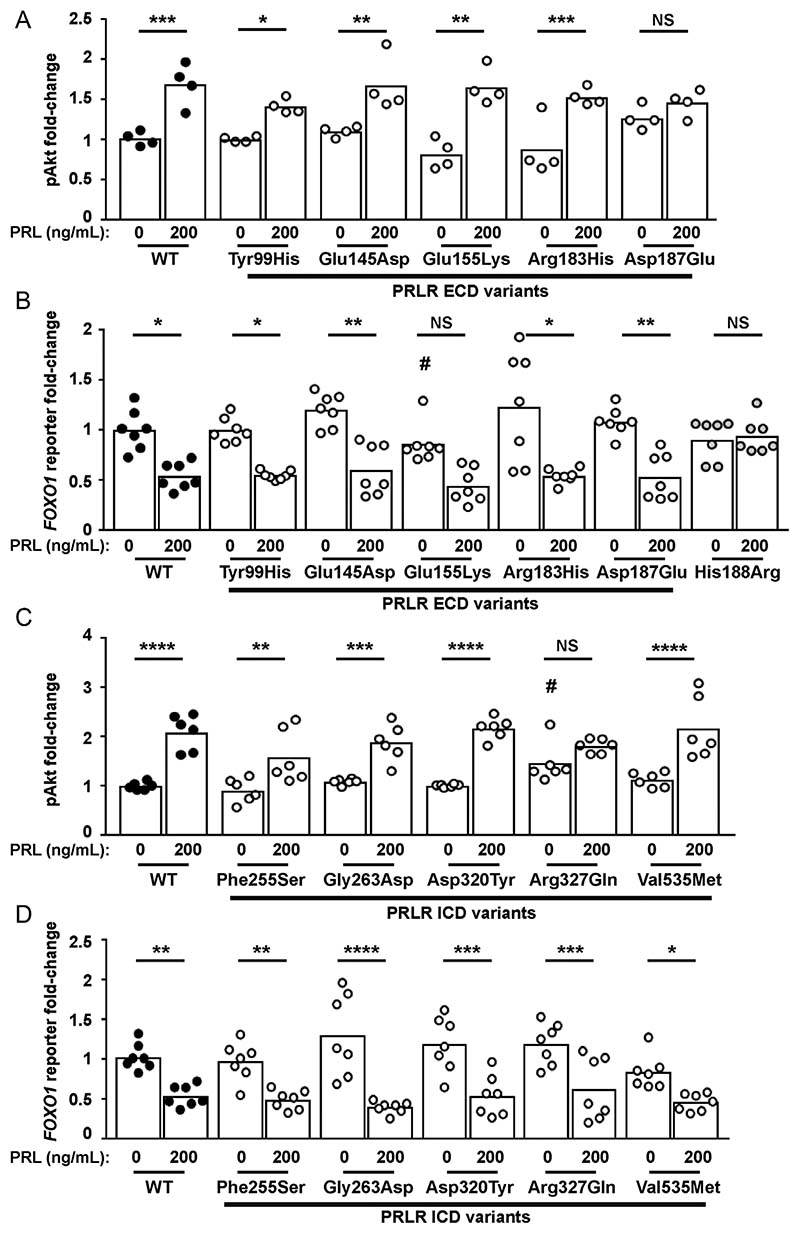
Functional characterisation of the Akt signalling pathway by PRLR rare variants. (A) pAkt responses following PRL treatment in cells transfected with wild-type (WT) or the five extracellular domain (ECD) variant PRLRs. Asp187Glu had impaired pAkt responses when compared to WT-expressing cells. (B) *FOXO1* luciferase reporter activity in cells transfected with WT, mutant His188Arg, or the five ECD variant PRLRs. Addition of 200 ng/mL PRL reduces *FOXO1* luciferase activity in WT and four variant cell lines. However, no response was observed in His188Arg cells and Glu155Lys had reduced basal *FOXO1* activity and impaired PRL-induced responses. (C) pAkt responses following PRL treatment in cells transfected with WT or the five intracellular domain (ICD) variant PRLRs. Arg327Gln-expressing cells had elevated basal pAkt activity. (D) *FOXO1* luciferase activity in cells transfected with WT or the five ICD variant PRLRs. Addition of 200 ng/mL prolactin to WT and variant PRLRs reduces *FOXO1* luciferase activity similarly in all cell lines. Data in all panels were expressed relative to WT cells treated with 0 ng/ mL PRL. Mean from 4 to 6 independent assays for pAkt assays. Mean (panel D) or median (panel B) from seven independent assays for luciferase reporter assays. Statistical analyses performed by one-way ANOVA with Sidak’s or Dunnett’s multiple comparisons tests for panels A, C, and D. Statistical analysis performed by Kruskal–Wallis test with Dunn’s multiple comparisons tests for panel B. Comparisons show 0 vs 200 nM PRL (asterisks) or between WT and variant (#). **** *P* < 0.0001, *** *P* < 0.001, ** *P* < 0.01, * *P* < 0.05, NS, not significant.

PRLR can also signal by the Akt pathway, and we have previously demonstrated that some PRLR rare variants affect signalling by this pathway (Gorvin *et al.* 2018b). To assess the effects of the PRLR variants on Akt signalling, we investigated PRL-induced responses of phospho-Akt by AlphaScreen analysis and luciferase reporter activity by the Akt-target gene *FOXO1* (Essaghir *et al.* 2009). Exposure of four of the PRLR ECD variants (Tyr99His, Glu145Asp, Glu155Lys, Arg183His) to 200 ng/mL PRL led to an increase in pAkt activity, which was not significantly different from that observed in WT PRLR expressing cells (Fig. 7A, Table 2). However, cells expressing the Asp187 Glu rare variant were unable to induce increases in p-Akt in response to 200 ng/mL PRL (Fig. 7A).

Akt phosphorylates FOXO proteins, resulting in their exclusion from the nucleus and subsequent degradation. Thus, PRL activation of the PRLR, which induces increases in Akt signalling, will reduce FOXO1 transcription (Essaghir *et al.* 2009). Assessment of *FOXO1* luciferase activity in cells expressing the WT or PRLR ECD variants showed that all cells could reduce *FOXO1* luciferase reporter activity in response to 200 ng/mL PRL (Fig. 7B). However, cells expressing the Glu155Lys variant had lower basal expression of *FOXO1* luciferase activity (Fig. 7B). In contrast, the His188Arg mutant, which has previously been shown to reduce pAkt activity (Gorvin *et al.* 2018b), was unable to reduce *FOXO1* luciferase reporter activity following exposure to 200 ng/mL PRL (Fig. 7B), consistent with impaired pAkt activity.

Assessment of the ICD PRLR rare variants showed that four variants (Phe255Ser, Gly263Asp, Asp320Tyr, Val535Met) had similar PRL-induced pAkt responses to WT PRLR-expressing cells (Fig. 7C, Table 2). However, the Arg327Gln variant did not increase pAkt in response to 200 ng/mL PRL. This may have been a consequence of constitutively high basal pAkt concentrations in Arg327Gln-expressing cells (Fig. 7C, Table 2). None of the five ICD variants had a significant effect on PRL-induced FOXO1 responses compared to the wild-type PRLR (Fig. 7D).

### Effect of the PRLR rare variants on cell viability and apoptosis

**Figure 8:**
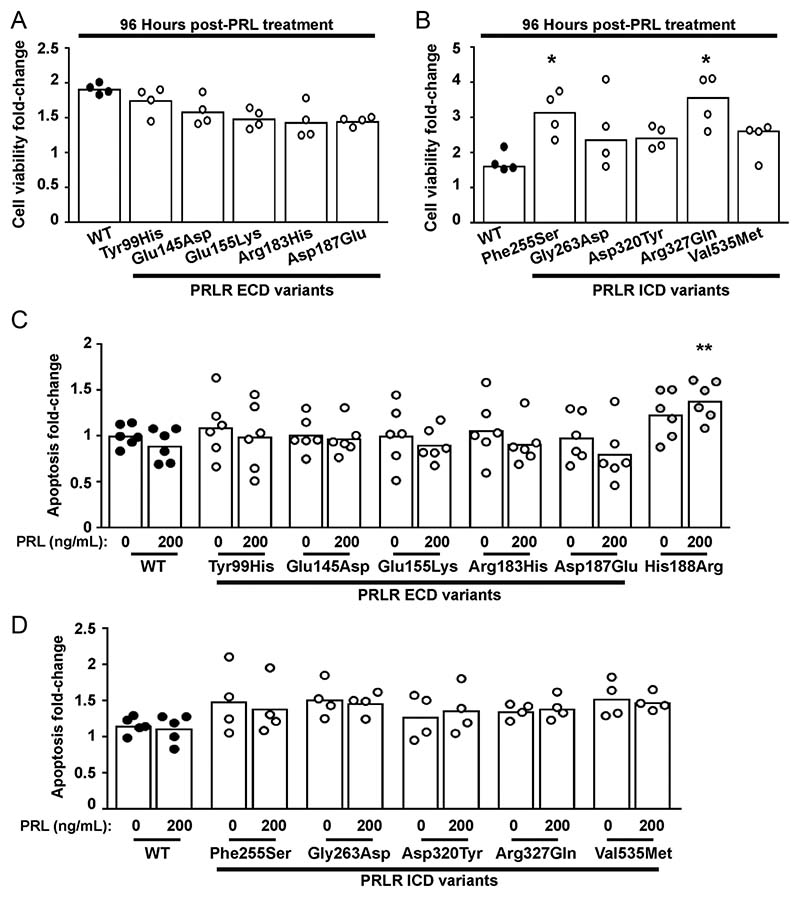
Effect of the PRLR rare variants on cell viability and apoptosis. (A-B) Effect of PRL (200 ng/mL) on viability in cells expressing WT, or (A) the extracellular domain (ECD) or (B) the intracellular domain (ICD) variant PRLRs. Cell viability was increased in cells expressing the Phe255Ser and Arg327Gln variant PRLRs at 96 h post-treatment with PRL, when compared to WT cells. (C-D) Effect of PRL (200 ng/mL) on apoptosis in cells expressing WT, mutant His188Arg, or (C) the ECD or (D) the ICD variant PRLRs. Each point shows one biological replicate (derived from the mean of four technical replicates) performed on independent occasions. Statistical analyses performed by one-way ANOVA with Dunnett’s multiple comparisons tests for panels A, C, and D. Statistical analysis performed by Kruskal–Wallis test with Dunn’s multiple comparisons tests for panel B. Comparisons show WT vs variant (asterisks). **** *P* < 0.0001, *** *P* < 0.001, ** *P* < 0.01, * *P* < 0.05.

Both the STAT5 and Akt signalling pathways lead to transcription of target genes that regulate proliferation and cell survival (Fresno Vara *et al.* 2000, Amaral *et al.* 2004, Brooks 2012), and previous studies have demonstrated that the Asn492Ile PRLR variant increases proliferation, while the His188Arg mutation increases apoptosis (Gorvin *et al.* 2018b). We therefore assessed the effect of the ten PRLR rare variants on cell viability using the CellTiter Blue assay and on apoptosis using a Caspase-Glo-3/7 assay (Gorvin *et al.* 2018b). This demonstrated that all five ECD variants (Tyr99His, Glu145Asp, Glu155Lys, Arg183His, Asp187Glu) and three ICD variants (Gly263Asp, Asp320Tyr Val535Met) had a similar effect on cell viability when compared to cells expressing WT PRLR, following exposure to 200 ng/mL PRL for 96 h (Fig. 8A and B, Table 2). The ICD Phe255Ser and Arg327Gln PRLR variants were associated with significantly increased numbers of viable cells after 96 h of PRL treatment. Assessment of apoptosis was performed in WT and variant PRLR expressing cells after 96 h of exposure to 200 ng/mL PRL. The ECD His188Arg loss-of-function mutation increased apoptosis in cells treated with 200 ng/mL PRL (Fig. 8C), consistent with our previous report (Gorvin *et al.* 2018b). However, none of the other ECD (Fig. 8C) or ICD (Fig. 8D) PRLR variants had a significant effect on apoptosis. Therefore, both loss-of-function and gain-of-function mutations in the PRLR increased the number of viable cells, although none of these investigated rare variants affected apoptosis.

## Discussion

Evaluating the clinical significance of rare coding variants within genes associated with Mendelian disorders and complex traits represents a significant challenge, and consequently, a range of *in silico* methods have been developed to facilitate the identification of potentially deleterious variants resulting in altered protein function. Our analysis of PRLR variants demonstrated that *in silico* tools could not accurately predict those that affected PRLR function (Table 2). This is consistent with previous studies that have shown that algorithms are only 65–80% accurate in predicting known disease variants (Thusberg *et al.* 2011) as pathogenic and often, over-predict missense changes as deleterious, while they are unreliable in predicting variants with milder effects (Choi *et al.* 2012). As such, the American College of Medical Genetics and Genomics (ACMG) recommends that protein prediction software is not used as the sole source of information to make clinical decisions (Richards *et al.* 2015). For the PRLR, only one variant, Phe255Ser, was correctly predicted deleterious in all four *in silico* methods (Table 1 and 2); while the gain-of-function Arg327Gln variant was predicted benign in three of four tools examined. This is consistent with previous studies of missense variants in the *ABCC8, GCK,* and *KCNJ11* genes that showed SIFT and Polyphen to be better at predicting inactivating than gain-of-function mutations (Flanagan *et al.* 2010). Furthermore, most *in silico* tools use the evolutionary conservation of the affected residue as a parameter to predict deleteriousness/pathogenicity (Richards *et al.* 2015). Such reliance on evolutionary conservation may be poorly predictive for PRLR variants, as the receptor has a specific role in lactation, and thus a lack of conservation with non-lactating species may be unimportant and could account for the poor predictive capability of *in silico* tools for PRLR.

Our functional studies identified five of ten PRLR germline variants that were associated with altered signalling, and these variants were all located in regions of the PRLR that have known receptor functions (Table 2). Thus, the three ECD variants that reduced PRLR function were predicted to affect ligand binding, receptor activation, and homodimerisation, while the ICD variants are located close to the JAK2-binding site and a known degradation motif (Fig. 1, 2 and 3). Examination of the ExAc/GnomAD databases identified significantly fewer ECD variants than predicted to occur in a region of this size and that there are more singleton variants (i.e. those identified in a single individual), which have previously been reported to have a higher probability of being functionally damaging and typically have occurred recently in evolutionary terms (Tennessen *et al.* 2012). These findings indicate that the ECD may be less tolerant of genetic variation, due to its critical roles in ligand binding and receptor activation, and that identification of variants in known functional domains is a reasonable predictor of possible pathogenicity. It is of note that several PRLR variants from other regions had similar responses to WT PRLR or were associated with, at most, modest effects on receptor function that may only be identified at supra-physiological concentrations of PRL (e.g. pSTAT5 responses for Gly263Asp and Val535Met).

The Glu155Lys variant is predicted to disrupt a contact formed across the homodimer interface and was associated with a partial loss of function for both STAT5 and pAkt signalling pathways (Fig. 2, 5 and 7, Table 2). However, analysis of protein expression in Glu155Lys-expressing cells showed no discernible difference in PRLR dimer or monomer concentrations (Fig. 4), indicating that loss of this homodimeric interaction is not sufficient to impair dimer formation. However, the partial loss of function associated with this Glu155Lys variant indicates that this residue may have an important role in facilitating conformational changes across the dimer interface that are necessary for receptor activation. The Glu155 residue lies in proximity to the WSXWS motif, a highly conserved motif of cytokine receptors, that holds the receptor in an ‘off-state’, until ligand interaction occurs, inducing formation of a Trp-Arg ladder to activate the receptor (Dagil *et al.* 2012). The Glu155Lys variant may disrupt these conformational changes, with a consequent reduction in signalling as observed in functional studies (Table 2). The elucidation of the full-length PRLR structure in the active and inactive states could help resolve whether the Glu155Lys variant has such functional effects.

Within the ICD, the Phe255 residue is located in the region between the two JAK2-binding sites, within a series of residues that are unnecessary for JAK2 phosphorylation but critical for downstream transcription of the beta-casein reporter gene (Lebrun *et al.* 1995, Ali & Ali 1998). We therefore hypothesise that Phe255 is involved in interaction with downstream signalling partners of PRLR that are necessary for pSTAT5 signalling but not for pAkt signalling, which was unaffected by the variant (Fig. 6 and 7, Table 2). Interestingly, the Phe255Ser variant was associated with increased numbers of viable cells, which we have previously observed for the gain-of-function Asn492Ile ICD variant (Gorvin *et al.* 2018b), and showed for the activating Arg327Gln variant in this study (Fig. 8, Table 2). Thus, both inactivating and activating ICD PRLR variants may be associated with increased cell survival and proliferation. It is likely that increased cell survival by the three receptor variants involves different mechanisms. The Asn492Ile variant had no effect on PRL-induced pSTAT5 signalling but increased Akt signalling, which could be rectified by treatment with a PI3K inhibitor (Gorvin *et al.* 2018b). Thus, it is likely this variant increases cell survival by activating the Akt-PI3K signalling pathway. The Arg327Gln variant does not increase PRL-induced Akt signalling but has constitutive Akt activity (Fig. 7, Table 2), which may contribute to increased numbers of viable cells. Additionally, the Arg327Gln variant significantly increases STAT5 signalling, which has previously been shown to promote mammary cell proliferation (Iavnilovitch *et al.* 2002). Thus, proliferation may be increased by both enhanced PRL-induced STAT5 signalling and constitutive Akt signalling. Furthermore, the Arg327 residue lies within the PRLR degradation motif (Plotnikov *et al.* 2009) and the Arg327Gln variant may impair receptor degradation. However, Western blot analyses did not show enhanced protein expression, indicating that Arg327Gln is unlikely to affect protein turnover. The mechanism by which the Phe255Ser variant increases cell viability is unknown. It is possible that this residue enhances binding of negative regulators of PRLR signalling, such as the suppressors of cytokine signalling (SOCS) proteins (Tomic *et al.* 1999), or may activate signalling pathways that are yet to be identified. Examination of proteins that regulate the cell cycle, including expression of cyclin D1 and transcription factors such as c-Myc, which are controlled by prolactin-mediated JAK-STAT and Akt signalling (Brockman *et al.* 2002, Acosta *et al.* 2003), could provide more insights into the different effects of PRLR variants on cell survival. The use of pathway-specific inhibitors, such as the Akt1/2 inhibitor previously used to examine the prolactinoma-associated Asn492Ile PRLR variant, may be required to further elucidate these mechanisms. Moreover, studies of additional PRLR ICD variants within the LID1 region may identify other residues with similar effects on signalling and proliferation.

Although these studies identified several inactivating PRLR variants, some activity was retained by all the variants, in contrast to the hyperprolactinaemia-associated His188Arg variant (Newey *et al.* 2013), which abolishes signalling. This is in keeping with the observation that the His188 residue occurs within the high-affinity ligand-binding interface and has a functional role in ligand binding and receptor activation (Kulkarni *et al.* 2010). The retention of some signalling activity may also explain why only the His188Arg variant is associated with enhanced apoptosis (Gorvin *et al.* 2018b). It is unclear whether the partial loss-of-activity associated with the PRLR variants examined in this study would affect PRLR physiological activities, as all the variants have been identified in the heterozygous state. Previous *in vitro* studies of the Pro269Leu PRLR variant identified in a compound heterozygote individual with hyperprolactinaemia showed that the variant impaired STAT5 phosphorylation but had no effect on STAT5 signalling when expressed with WT PRLR (Kobayashi *et al.* 2018). Thus, it is possible that the ten variants characterised in this study may have minimal effect on PRLR function in heterozygous individuals, unless expressed with other PRLR variants. Further investigation of the inactivating and activating PRLR variants in large well-characterised populations is required to determine their physiological consequences.

This study and our previous analysis of PRLR variants associated with prolactinoma demonstrated that both JAK-STAT and Akt signalling can be impaired by genetic variants of the receptor (Gorvin *et al.* 2018a). The PRLR has also been described to activate other signalling pathways including Ras–Raf-mediated mitogen-activated protein kinase (MAPK) signalling (Bole-Feysot *et al.* 1998) and can activate Src family kinases independently of JAK2 phosphorylation (Fresno Vara *et al.* 2000) to increase focal-adhesion kinase/MAPK and PI3K-Akt signalling, upregulate c-Myc and cyclin d1 mRNA expression, enhance cell proliferation, and accelerate receptor internalisation (Acosta *et al.* 2003, Piazza *et al.* 2009). It is possible that the PRLR variants studied in this manuscript may also affect these other signalling pathways and the observed effects on Akt could be mediated by JAK2-independent Src signalling. Src family kinases and MAPK signalling proteins are expressed in HEK293 cells (Della Rocca *et al.* 1997), and future studies of PRLR variants could expand the screening pipeline to include examination of these proteins.

In summary, these studies give further insight into PRLR structure–function and highlight that rare PRLR variants are associated with alterations in receptor signalling. Future studies of rare coding variants will require a combination of molecular, *in vivo,* and epidemiological approaches to appropriately classify the significance of such variants.

## Figures and Tables

**Table 1:** PRLR rare variants predicted to be potentially deleterious/pathogenic in the GnomAD database.

	Amino acid change	Residue	Protein predictions^a^				Evolutionary conservation^b^	Predicted structural effect^c^
			SIFT	Polyphen	MutationTaster	REVEL		
**ECD D1**	Pro>Leu	3 (27)	Tolerated	Probably damaging	Disease causing	Benign	4	No change
	Pro>His	3 (27)	Deleterious	Probably damaging	Polymorphism	Benign	4	No change
	Lys>Asn	6 (30)	Deleterious	Probably damaging	Disease causing	Benign	4	Loss of contact with Asp91
	Pro>Ser	7 (31)	Deleterious	Probably damaging	Disease causing	Disease causing	4	Gains contact with Val79
	Arg>His	13 (37)	Deleterious	Probably damaging	Disease causing	Disease causing	4	No change
	Glu>Lys	18 (42)	Deleterious	Probably damaging	Disease causing	Benign	4	No change
	Thr>Ile	21 (45)	Deleterious	Probably damaging	Disease causing	Disease causing	4	No change
	Gly>Arg	30 (54)	Deleterious	Probably damaging	Disease causing	Benign	4	Gains contact with Asn83
	Gly>Arg	31 (55)	Deleterious	Probably damaging	Disease causing	Disease causing	4	No change
	Thr>Ser	34 (58)	Deleterious	Benign	Disease causing	Benign	4	No change
	His>Arg	49 (73)	Deleterious	Possibly damaging	Polymorphism	Benign	2	No change
	Cys>Tyr	51 (75)	Deleterious	Probably damaging	Disease causing	Disease causing	4	Gains contact with Phe63
	Gly>Asp	58 (82)	Deleterious	Probably damaging	Disease causing	Benign	4	No change
	Asn>Ser	60 (84)	Deleterious	Possibly damaging	Disease causing	Benign	4	No change
	Ile>Met	76 (100)	Tolerated	Possibly damaging	Polymorphism	Benign	3	No change
	Gln>His	84 (108)	Deleterious	Possibly damaging	Disease causing	Benign	3	No change
	Met>Ile	85 (109)	Tolerated	Probably damaging	Disease causing	Benign	4	No change
	Ser>Arg	88 (112)	Deleterious	Possibly damaging	Polymorphism	Benign	4	Gains contact with Ser87 and loses contact with Pro4
	Val>Leu	95 (119)	Deleterious	Possibly damaging	Disease causing	Benign	4	No change
	Asp>Glu	96 (120)	Tolerated	Probably damaging	Disease causing	Benign	3	No change
	Val>Met	97 (121)	Deleterious	Probably damaging	Disease causing	Benign	4	No change
	**Tyr>His**	**99 (123)**	**Deleterious**	**Probably damaging**	**Disease causing**	**Benign**	**4**	**Loses contact with PRL**
	Ile>Val	100 (124)	Deleterious	Probably damaging	Disease causing	Benign	4	No change
**ECD D2**	Leu>Met	109 (133)	Deleterious	Possibly damaging	Polymorphism	Benign	4	No change
	Pro>Ala	129 (153)	Deleterious	Possibly damaging	Disease causing	Likely benign	4	No change
	Pro>Leu	129 (153)	Deleterious	Possibly damaging	Disease causing	Disease causing	4	No change
	Thr>Met	141 (165)	Deleterious	Benign	Disease causing	Benign	3	Loses contact with Asp187
	Tyr>Asn	144 (168)	Deleterious	Probably damaging	Disease causing	Disease causing	4	No change
	**Glu>Asp**	**145 (169)**	**Deleterious**	**Probably damaging**	**Disease causing**	**Benign**	**4**	**Loses 3 of 4 contacts with Arg183, gains contact with Ile146**
	Arg>Gln	147 (171)	Tolerated	Benign	Disease causing	Benign	4	No change
	Glu>Lys	151 (175)	Deleterious	Benign	Disease causing	Benign	3	No change
	**Glu>Lys**	**155 (179)**	**Deleterious**	**Possibly damaging**	**Disease causing**	**Benign**	**3**	**Loses contact with Lys114 on opposite PRLR protomer**
	Trp>Leu	156 (180)	Deleterious	Possibly damaging	Disease causing	Benign	4	No change
	Ile>Val	169 (193)	Tolerated	Benign	Disease causing	Likely benign	2	No change
	Leu>Val	172 (196)	Tolerated	Benign	Disease causing	Benign	4	No change
	Gly>Glu	175 (199)	Deleterious	Probably damaging	Disease causing	Benign	4	No change
	Leu>Phe	179 (203)	Tolerated	Benign	Disease causing	Benign	4	No change
	Val>Ile	180 (204)	Tolerated	Benign	Disease causing	Benign	4	No change
	**Arg>His**	**183 (207)**	**Tolerated**	**Probably damaging**	**Disease causing**	**Benign**	**4**	**Loses 2 of 4 contacts with Glu145; loses contact with Ala193 of WSxWS motif**
	**Asp>Glu**	**187 (211)**	**Deleterious**	**Possibly damaging**	**Disease causing**	**Benign**	**4**	**Loses contact with His188**
	Gln>Arg	201 (225)	Deleterious	Benign	Polymorphism	Benign	2	No change
	Asp>Asn	205 (229)	Tolerated	Benign	Disease causing	Benign	4	No change
**TMD**	Ala>Gly	222 (246)	Deleterious	Benign	Disease causing	Benign	4	No change
	Ile>Thr	227 (251)	Tolerated	Possibly damaging	Disease causing	Disease causing	4	No change
	Trp>Cys	230 (254)	Deleterious	Possibly damaging	Disease causing	Disease causing	4	No change
	Val>Ala	232 (256)	Deleterious	Benign	Disease causing	Disease causing	4	No change
	Val>Met	232 (256)	Tolerated	Benign	Possibly damaging	Benign	4	No change
**ICD**	Lys>Arg	235 (259)	Deleterious	Probably damaging	Disease causing	Disease causing	4	Unknown
	Gly>Ser	236 (260)	Tolerated	Possibly damaging	Disease causing	Benign	4	LID1, Box 1
	Met>Leu	239 (263)	Tolerated	Benign	Possibly damaging	Benign	4	LID1, Box 1
	Cys>Arg	242 (266)	Tolerated	Probably damaging	Disease causing	Disease causing	4	LID1, Box 1
	Ile>Val	243 (267)	Tolerated	Benign	Disease causing	Likely benign	4	LID1, Box 1
	Pro>Leu	245 (269)	Deleterious	Probably damaging	Disease causing	Disease causing	4	LID1, Box 1
	Pro>Ser	246 (270)	Deleterious	Probably damaging	Disease causing	Disease causing	4	LID1, Box 1
	Lys>Asn	251 (275)	Deleterious	Probably damaging	Disease causing	Disease causing	4	LID1, Box 1
	Lys>Arg	253 (277)	Tolerated	Probably damaging	Disease causing	Benign	4	LID1
	Lys>Glu	253 (277)	Deleterious	Probably damaging	Disease causing	Disease causing	4	LID1
	**Phe>Ser**	**255 (279)**	**Deleterious**	**Probably damaging**	**Disease causing**	**Disease causing**	**4**	**LID1**
	Asp>Ala	256 (280)	Deleterious	Probably damaging	Disease causing	Disease causing	4	LID1
	Leu>Trp	260 (284)	Deleterious	Probably damaging	Disease causing	Disease causing	4	LID1
	Gly>Ser	263 (287)	Deleterious	Probably damaging	Disease causing	Disease causing	4	LID1
	**Gly>Asp**	**263 (287)**	**Deleterious**	**Probably damaging**	**Disease causing**	**Disease causing**	**4**	**LID1**
	Lys>Gln	264 (288)	Deleterious	Probably damaging	Disease causing	Benign	4	LID1
	Ser>Cys	265 (289)	Deleterious	Probably damaging	Disease causing	Disease causing	4	LID1
	Glu>Gln	266 (290)	Deleterious	Probably damaging	Disease causing	Benign	4	LID1
	Ser>Thr	270 (294)	Tolerated	Benign	Disease causing	Benign	3	LID1
	Ser>Arg	270 (294)	Deleterious	Benign	Disease causing	Benign	3	LID1
	Ala>Val	271 (295)	Deleterious	Possibly damaging	Disease causing	Benign	4	LID1
	Leu>Ser	272 (296)	Deleterious	Possibly damaging	Disease causing	Disease causing	4	LID1
	Gly>Val	273 (297)	Tolerated	Probably damaging	Disease causing	Benign	3	LID1
	Asp>Tyr	276 (300)	Deleterious	Possibly damaging	Disease causing	Benign	4	LID1
	Pro>His	278 (302)	Deleterious	Probably damaging	Disease causing	Disease causing	4	LID1
	Ser>Phe	281 (305)	Deleterious	Probably damaging	Disease causing	Disease causing	4	LID1
	Ser>Tyr	281 (305)	Deleterious	Probably damaging	Disease causing	Disease causing	4	LID1
	Asp>Asn	285 (309)	Deleterious	Probably damaging	Disease causing	Disease causing	4	LID1
	Asp>Glu	285 (309)	Tolerated	Benign	Disease causing	Likely benign	4	LID1
	Val>Ile	293 (317)	Tolerated	Possibly damaging	Disease causing	Benign	4	LID1, Box 2
	Asp>Glu	294 (318)	Tolerated	Possibly damaging	Polymorphism	Benign	4	LID1, Box 2
	Asp>Asn	298 (322)	Deleterious	Benign	Disease causing	Benign	4	LID1
	Leu>Ile	301 (325)	Deleterious	Possibly damaging	Disease causing	Benign	4	
	Met>Ile	302 (326)	Tolerated	Benign	Possibly damaging	Benign	4	
	Pro>Arg	316 (340)	Deleterious	Possibly damaging	Polymorphism	Benign	4	
	**Asp>Tyr**	**320 (344)**	**Deleterious**	**Probably damaging**	**Polymorphism**	**Benign**	**4**	
	**Arg>Gln**	**327 (351)**	**Tolerated**	**Benign**	**Disease causing**	**Benign**	**2**	**Degradation motif**
	Arg>Pro	327 (351)	Deleterious	Probably damaging	Disease causing	Disease causing	2	Degradation motif
	Arg>Trp	327 (351)	Deleterious	Probably damaging	Disease causing	Disease causing	2	Degradation motif
	Asp>Asn	331 (355)	Deleterious	Probably damaging	Disease causing	Benign	4	
	Pro>Arg	333 (357)	Deleterious	Possibly damaging	Disease causing	Benign	2	
	Ser>Phe	334 (358)	Tolerated	Probably damaging	Disease causing	Benign	4	
	Cys>Tyr	340 (364)	Tolerated	Benign	Disease causing	Benign	4	
	Glu>Lys	342 (366)	Deleterious	Possibly damaging	Dsease causing	Benign	4	
	Pro>Thr	353 (377)	Tolerated	Probably damaging	Polymorphism	Benign	4	LID2
	Glu>Gly	376 (400)	Deleterious	Possibly damaging	Polymorphism	Benign	2	LID2
	Tyr>Cys	381 (405)	Tolerated	Benign	Disease causing	Benign	2	LID2
	Gly>Glu	386 (410)	Tolerated	Possibly damaging	Polymorphism	Benign	2	
	Gln>Glu	396 (420)	Deleterious	Probably damaging	Polymorphism	Benign	2	
	Arg>Ile	403 (427)	Deleterious	Possibly damaging	Polymorphism	Benign	4	
	Asp>Val	411 (435)	Deleterious	Possibly damaging	Disease causing	Disease causing	4	
	Asp>Tyr	411 (435)	Deleterious	Possibly damaging	Polymorphism	Disease causing	4	
	Cys>Arg	413 (437)	Tolerated	Possibly damaging	Polymorphism	Benign	4	
	Cys>Tyr	413 (437)	Tolerated	Possibly damaging	Polymorphism	Benign	4	
	Thr>Pro	425 (449)	Tolerated	Possibly damaging	Polymorphism	Likely benign	2	
	Asp>Asn	463 (487)	Tolerated	Benign	Disease causing	Benign	2	
	Gln>His	472 (496)	Tolerated	Possibly damaging	Polymorphism	Benign	4	
	Lys>Glu	481 (505)	Tolerated	Possibly damaging	Polymorphism	Benign	3	
	Asp>Asn	484 (508)	Deleterious	Possibly damaging	Disease causing	Disease causing	3	
	Val>Met	486 (510)	Deleterious	Probably damaging	Disease causing	Disease causing	4	
	Glu>Ala	487 (511)	Deleterious	Probably damaging	Disease causing	Disease causing	4	
	Glu>Lys	487 (511)	Deleterious	Probably damaging	Disease causing	Disease causing	4	
	1 le>P he	488 (512)	Deleterious	Probably damaging	Disease causing	Benign	4	
	Ile>Thr	488 (512)	Deleterious	Probably damaging	Disease causing	Disease causing	4	
	His>Tyr	489 (513)	Deleterious	Probably damaging	Disease causing	Disease causing	4	
	Gly>Val	495 (519)	Deleterious	Probably damaging	Disease causing	Benign	4	
	Leu>Phe	497 (521)	Deleterious	Probably damaging	Polymorphism	Benign	4	
	Leu>Arg	500 (524)	Deleterious	Possibly damaging	Polymorphism	Disease causing	2	
	Pro>Gln	501 (525)	Deleterious	Probably damaging	Disease causing	Benign	4	
	Pro>Thr	501 (525)	Tolerated	Possibly damaging	Disease causing	Benign	4	
	Pro>Leu	501 (525)	Tolerated	Benign	Disease causing	Benign	4	
	Arg>Thr	504 (528)	Deleterious	Benign	Polymorphism	Benign	2	
	Lys>Asn	520 (544)	Deleterious	Probably damaging	Disease causing	Benign	4	
	Glu>Lys	521 (545)	Deleterious	Probably damaging	Disease causing	Disease causing	4	
	Lys>Glu	524 (548)	Deleterious	Possibly damaging	Disease causing	Disease causing	4	
	Asp>His	530 (554)	Deleterious	Probably damaging	Disease causing	Benign	4	
	Asp>Gly	530 (554)	Tolerated	Possibly damaging	Disease causing	Benign	4	
	Asn>Lys	531 (555)	Deleterious	Benign	Polymorphism	Benign	3	
	Asn>Ser	532 (556)	Tolerated	Benign	Disease causing	Benign	4	
	Leu>Val	534 (558)	Deleterious	Probably damaging	Disease causing	Disease causing	4	
	**Val>Met**	**535 (559)**	**Deleterious**	**Probably damaging**	**Disease causing**	**Disease causing**	**4**	
	Asp>His	539 (563)	Deleterious	Probably damaging	Polymorphism	Benign	3	
	Ala>Asp	542 (566)	Tolerated	Possibly damaging	Polymorphism	Benign	4	
	Glu>Lys	549 (573)	Deleterious	Possibly damaging	Polymorphism	Benign	4	LID3
	Glu>Gly	549 (573)	Deleterious	Possibly damaging	Polymorphism	Benign	4	LID3
	Ala>Asp	552 (576)	Deleterious	Benign	Polymorphism	Disease causing	4	LID3
	Lys>Arg	553 (577)	Tolerated	Possibly damaging	Polymorphism	Benign	4	LID3
	Asn>Lys	562 (586)	Tolerated	Possibly damaging	Polymorphism	Benign	2	LID3
	Asn>Tyr	562 (586)	Deleterious	Probably damaging	Polymorphism	Benign	2	LID3
	Leu>Pro	580 (604)	Tolerated	Probably damaging	Disease causing	Disease causing	3	LID3
	Asp>Tyr	586 (610)	Deleterious	Probably damaging	Disease causing	Disease causing	3	LID3
	Asp>Gly	589 (613)	Deleterious	Probably damaging	Disease causing	Disease causing	3	LID3
	Pro>Arg	590 (614)	Deleterious	Probably damaging	Disease causing	Disease causing	3	LID3
	Cys>Arg	592 (616)	Tolerated	Possibly damaging	Polymorphism	Disease causing	3	LID3

a Protein predictions were made using SIFT, Polyphen-2, MutationTaster, and REVEL;

b Evolutionary conservation was based on the PRLR ortholog sequence in four species (cow, dog, mouse, and rat) in comparison to the human protein. The score shows the number of species (out of four) in which the residue is conserved;

c Structural analyses were performed using the reported crystal structure of the ligand-bound homodimeric ECD and the NMR structure of the D2 domain (van Agthoven *et al.* 2010, Dagil *et al.* 2012) (Figs. 1, 2 and 3). The ten PRLR variants shown in bold are those that were functionally characterised.

**Table 2:** Summary of effects of rare variants on PRLR function.

	PRLR variant	Predicted pathogenicity			STAT5 signalling		PI3K/ Akt signalling		Cell viability	Apoptosis
		Prediction programs^a^	Evolutionary conservation^b^	Structural predictions	pSTAT5	CISH	pAkt	FOXO1		
	**WT**	0	N/A	N/A	++	++	++	++	++	++
	**His188Arg**	4	4	Affects PRL binding	-	-	-	-	++	+++
ECD	**Tyr99His**	3	4	Affects PRL binding	+	+	++	++	++	++
ECD	**Glu145Asp**	3	4	Loss and gain of contacts	++	++	++	++	++	++
ECD	**Glu155Lys**	3	3	Disrupts homodimer region	+	+	++	+	++	++
ECD	**Arg183His**	2	4	Loses contact with WSxWS motif	++	++	++	++	++	++
ECD	**Asp187Glu**	3	4	Loses contact with His188	-	+	+	++	++	++
ICD	**Phe255Ser**	4	4	LID1, close to JAK2 binding site	-	+	++	++	+++	++
ICD	**Gly263Asp**	4	4	LID1	++	++	++	++	++	++
ICD	**Asp320Tyr**	2	4	Unknown	++	++	++	++	++	++
ICD	**Arg327Gln**	1	2	Degradation motif	+++	+++	+ *	++	+++	++
ICD	**Val535Met**	4	4	Unknown	++	++	++	++	++	++

a A score out of four based on protein predictions using SIFT, Polyphen-2, MutationTaster, and REVEL is given. If the variant was predicted to be probably damaging or damaging/pathogenic, it was classified as affected;

b Evolutionary conservation was based on the PRLR ortholog sequence in four species (cow, dog, mouse, and rat) in comparison to the human protein. The score shows the number of species in which the residue is conserved out of 4;

+++ Gain of function;

++ Normal;

+ Impaired;

- Abolished.;

* Arg327Gln has significantly increased basal Akt activity, which likely accounts for the impaired PRL-induced activity. N/A, not applicable.
